# *N*-(4-Chloro-3-nitro­phen­yl)maleamic acid

**DOI:** 10.1107/S1600536812008021

**Published:** 2012-02-29

**Authors:** U. Chaithanya, Sabine Foro, B. Thimme Gowda

**Affiliations:** a Department of Chemistry, Mangalore University, Mangalagangotri 574 199, Mangalore, India; b Institute of Materials Science, Darmstadt University of Technology, Petersenstrasse 23, D-64287 Darmstadt, Germany

## Abstract

In the mol­ecule of the title compound, C_10_H_7_ClN_2_O_5_, the acyclic C=C double bond is *cis* configured. The C=O and O—H bonds of the acid group are in a relatively rare *anti* position to each other, due to the donation of intramolecular hydrogen bond to the amide by the carboxyl group. The nitro group is significantly twisted [dihedral angle = 66.9 (3)°] out of the plane of the remaining atoms, which are almost coplanar (r.m.s. deviation for non-H atoms except the nitro group = 0.202 Å). In the crystal, N—H⋯O hydrogen bonds link the mol­ecules into zigzag chains running along the *b* axis.

## Related literature

For our studies of the effects of substituents on the structures and other aspects of *N*-(ar­yl)amides, see: Gowda *et al.* (2000 ▶, 2003 ▶); Chaithanya *et al.* (2012 ▶); *N*-(ar­yl)methane­sulfonamides, see: Gowda *et al.* (2007 ▶); *N*-chloro­aryl­amides, see: Jyothi & Gowda (2004 ▶) and *N*-bromo­aryl­sulfonamides, see: Usha & Gowda (2006 ▶).
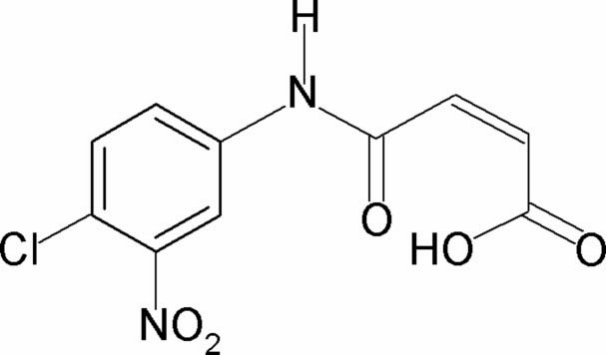


## Experimental

### 

#### Crystal data

C_10_H_7_ClN_2_O_5_
*M* *_r_* = 270.63
Monoclinic, 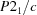

*a* = 9.7187 (9) Å
*b* = 13.596 (1) Å
*c* = 8.4990 (9) Å
β = 99.64 (1)°
*V* = 1107.16 (18) Å^3^
*Z* = 4
Mo *K*α radiation
μ = 0.36 mm^−1^
*T* = 293 K
0.42 × 0.12 × 0.06 mm

#### Data collection

Oxford Xcalibur diffractometer with a Sapphire CCD detector
Absorption correction: multi-scan (*CrysAlis RED*; Oxford Diffraction, 2009 ▶) *T*_min_ = 0.863, *T*_max_ = 0.979
4360 measured reflections
2243 independent reflections
1540 reflections with *I* > 2σ(*I*)
*R*_int_ = 0.025

#### Refinement

*R*[*F* ^2^ > 2σ(*F* ^2^)] = 0.063
*wR*(*F* ^2^) = 0.180
*S* = 0.97
2243 reflections
169 parameters
2 restraints
H atoms treated by a mixture of independent and constrained refinement
Δρ_max_ = 0.38 e Å^−3^
Δρ_min_ = −0.25 e Å^−3^

### 

Data collection: *CrysAlis CCD* (Oxford Diffraction, 2009 ▶); cell refinement: *CrysAlis RED* (Oxford Diffraction, 2009 ▶); data reduction: *CrysAlis RED*; program(s) used to solve structure: *SHELXS97* (Sheldrick, 2008 ▶); program(s) used to refine structure: *SHELXL97* (Sheldrick, 2008 ▶); molecular graphics: *PLATON* (Spek, 2009 ▶); software used to prepare material for publication: *SHELXL97*.

## Supplementary Material

Crystal structure: contains datablock(s) I, global. DOI: 10.1107/S1600536812008021/bt5827sup1.cif

Structure factors: contains datablock(s) I. DOI: 10.1107/S1600536812008021/bt5827Isup2.hkl

Supplementary material file. DOI: 10.1107/S1600536812008021/bt5827Isup3.cml

Additional supplementary materials:  crystallographic information; 3D view; checkCIF report

## Figures and Tables

**Table 1:** Hydrogen-bond geometry (Å, °)

*D*—H⋯*A*	*D*—H	H⋯*A*	*D*⋯*A*	*D*—H⋯*A*
O3—H3O⋯O1	0.82 (2)	1.67 (2)	2.494 (4)	174 (7)
N1—H1N⋯O2^i^	0.86 (2)	2.01 (2)	2.839 (4)	162 (4)

Symmetry code: (i) 
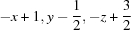
.

**Fig. 1.**
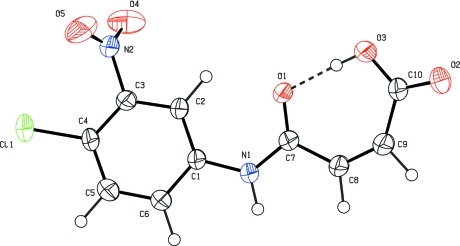
Molecular structure of the title compound, showing the atom labelling scheme and with displacement ellipsoids drawn at the 50% probability level.

**Fig. 2.**
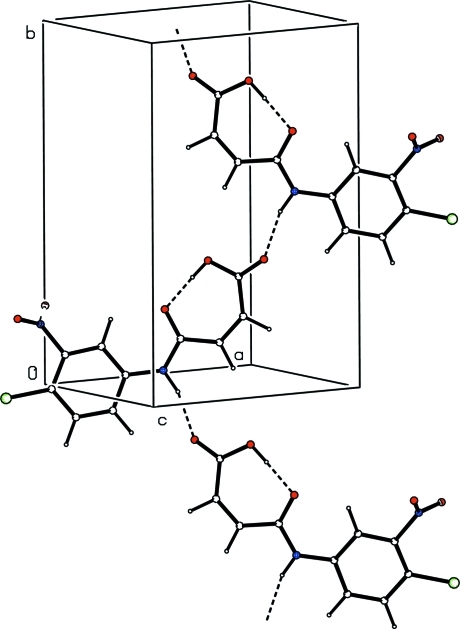
Molecular packing of the title compound with hydrogen bonding shown as dashed lines.

## supplementary crystallographic information

### Comment

As part of our studies on the substituent effects on the structures and other aspects of *N*-(aryl)-amides (Gowda *et al.*, 2000, 2003; Chaithanya *et al.*, 2012), *N*-(aryl)-methanesulfonamides (Gowda *et al.*, 2007); *N*-chloroarylsulfonamides (Jyothi & Gowda, 2004) and *N*-bromoarylsulfonamides (Usha & Gowda, 2006), in the present work, the crystal structure of *N*-(4-Chloro-3-nitrophenyl)maleamic acid has been determined (Fig. 1). The conformations of the N—H and the C═O bonds in the amide segment are *anti* to each other. The N—H bond is also *anti* to the *meta*–nitro group. Further, the conformation of the amide C═O is *anti* to the H atom on the adjacent –CH group, while the carboxyl C═O of the acid segment is *syn* to the adjacent –CH group. Furthermore, the C═O and O—H bond of the acid group are in relatively rare *anti* position to each other, due to the donation of hydrogen bond to the amide by the carboxyl group, similar to that observed in *N*-(3-Chloro-4-methylphenyl)maleamic acid (I) (Chaithanya *et al.*, 2012).

The dihedral angle between the phenyl ring and the amide group in the title compound is 11.52 (27)°, compared to the value of 6.55 (99)° in (I).

In the structure, the pairs of O—H···O and N—H···O intermolecular hydrogen bonds pack the molecules into zigzag chains (Table 1, Fig.2).

### Experimental

Maleic anhydride (0.025 mol) in toluene (25 ml) was treated dropwise with 4-chloro-3-nitroaniline (0.025 mol) also in toluene (20 ml) with constant stirring. The resulting mixture was stirred for about 30 min and set aside for an additional 30 min at room temperature for the completion of reaction. The mixture was then treated with dilute hydrochloric acid to remove the unreacted 4-chloro-3-nitroaniline. The resultant solid *N*-(4-Chloro-3-nitrophenyl)maleamic acid was filtered under suction and washed thoroughly with water to remove the unreacted maleic anhydride and maleic acid. It was recrystallized to constant melting point from ethanol. The purity of the compound was checked and characterized by its infrared spectra.

Rod like colorless single crystals of the title compound used in X-ray diffraction studies were grown in an ethanol solution by slow evaporation of the solvent (0.5 g in about 30 ml of ethanol) at room temperature.

### Refinement

The H atoms of the NH group and the OH group were located in a difference map and later restrained to the distance N—H = 0.86 (2) Å and O—H = 0.82 (2) Å, respectively. The other H atoms were positioned with idealized geometry using a riding model with the aromatic C—H = 0.93 Å and methylene C—H = 0.97 Å. All H atoms were refined with isotropic displacement parameters set at 1.2*U*_eq_.

### Crystal data

**Table:**

C_10_H_7_ClN_2_O_5_	*F*(000) = 552
*M**_r_* = 270.63	*D*_x_ = 1.624 Mg m^−^^3^
Monoclinic, *P*2_1_/*c*	Mo *K*α radiation, λ = 0.71073 Å
Hall symbol: -P 2ybc	Cell parameters from 1582 reflections
*a* = 9.7187 (9) Å	θ = 2.6–27.7°
*b* = 13.596 (1) Å	µ = 0.36 mm^−^^1^
*c* = 8.4990 (9) Å	*T* = 293 K
β = 99.64 (1)°	Rod, colourless
*V* = 1107.16 (18) Å^3^	0.42 × 0.12 × 0.06 mm
*Z* = 4	

### Data collection

**Table:**

Oxford Xcalibur diffractometer with a Sapphire CCD detector	2243 independent reflections
Radiation source: fine-focus sealed tube	1540 reflections with *I* > 2σ(*I*)
Graphite monochromator	*R*_int_ = 0.025
Rotation method data acquisition using ω and φ scans	θ_max_ = 26.4°, θ_min_ = 2.6°
Absorption correction: multi-scan (*CrysAlis RED*; Oxford Diffraction, 2009)	*h* = −12→6
*T*_min_ = 0.863, *T*_max_ = 0.979	*k* = −16→16
4360 measured reflections	*l* = −7→10

### Refinement

**Table:**

Refinement on *F*^2^	Primary atom site location: structure-invariant direct methods
Least-squares matrix: full	Secondary atom site location: difference Fourier map
*R*[*F*^2^ > 2σ(*F*^2^)] = 0.063	Hydrogen site location: inferred from neighbouring sites
*wR*(*F*^2^) = 0.180	H atoms treated by a mixture of independent and constrained refinement
*S* = 0.97	*w* = 1/[σ^2^(*F*_o_^2^) + (0.0665*P*)^2^ + 3.5725*P*] where *P* = (*F*_o_^2^ + 2*F*_c_^2^)/3
2243 reflections	(Δ/σ)_max_ = 0.034
169 parameters	Δρ_max_ = 0.38 e Å^−^^3^
2 restraints	Δρ_min_ = −0.25 e Å^−^^3^

### Special details

**Table:**

Experimental. CrysAlis RED (Oxford Diffraction, 2009) Empirical absorption correction using spherical harmonics, implemented in SCALE3 ABSPACK scaling algorithm.
Geometry. All e.s.d.'s (except the e.s.d. in the dihedral angle between two l.s. planes) are estimated using the full covariance matrix. The cell e.s.d.'s are taken into account individually in the estimation of e.s.d.'s in distances, angles and torsion angles; correlations between e.s.d.'s in cell parameters are only used when they are defined by crystal symmetry. An approximate (isotropic) treatment of cell e.s.d.'s is used for estimating e.s.d.'s involving l.s. planes.
Refinement. Refinement of *F*^2^ against ALL reflections. The weighted *R*-factor *wR* and goodness of fit *S* are based on *F*^2^, conventional *R*-factors *R* are based on *F*, with *F* set to zero for negative *F*^2^. The threshold expression of *F*^2^ > σ(*F*^2^) is used only for calculating *R*-factors(gt) *etc*. and is not relevant to the choice of reflections for refinement. *R*-factors based on *F*^2^ are statistically about twice as large as those based on *F*, and *R*-factors based on ALL data will be even larger.

### Fractional atomic coordinates and isotropic or equivalent isotropic displacement parameters (Å^2^)

**Table:**

	*x*	*y*	*z*	*U*_iso_*/*U*_eq_	
Cl1	−0.22417 (12)	−0.02257 (9)	0.06740 (14)	0.0526 (4)	
O1	0.3160 (3)	0.2207 (2)	0.5078 (4)	0.0543 (10)	
O2	0.6054 (3)	0.3654 (2)	0.8733 (4)	0.0527 (9)	
O3	0.4475 (4)	0.3576 (2)	0.6601 (4)	0.0582 (10)	
H3O	0.402 (6)	0.315 (3)	0.604 (6)	0.087*	
O4	−0.0092 (5)	0.2184 (3)	0.0224 (5)	0.0750 (12)	
O5	−0.1941 (4)	0.1979 (3)	0.1206 (6)	0.0852 (14)	
N1	0.3025 (3)	0.0549 (2)	0.5150 (4)	0.0353 (8)	
H1N	0.332 (5)	0.004 (2)	0.569 (5)	0.042*	
N2	−0.0772 (4)	0.1760 (3)	0.1083 (4)	0.0420 (9)	
C1	0.1800 (4)	0.0411 (3)	0.4016 (5)	0.0320 (9)	
C2	0.1145 (4)	0.1155 (3)	0.3048 (5)	0.0337 (9)	
H2	0.1534	0.1781	0.3068	0.040*	
C3	−0.0098 (4)	0.0945 (3)	0.2053 (5)	0.0331 (9)	
C4	−0.0696 (4)	0.0024 (3)	0.1939 (5)	0.0357 (10)	
C5	−0.0005 (5)	−0.0720 (3)	0.2858 (5)	0.0441 (11)	
H5	−0.0375	−0.1352	0.2787	0.053*	
C6	0.1233 (4)	−0.0536 (3)	0.3887 (5)	0.0416 (11)	
H6	0.1688	−0.1045	0.4494	0.050*	
C7	0.3599 (4)	0.1411 (3)	0.5669 (5)	0.0357 (9)	
C8	0.4800 (4)	0.1338 (3)	0.6985 (5)	0.0379 (10)	
H8	0.5099	0.0704	0.7281	0.046*	
C9	0.5505 (4)	0.2064 (3)	0.7794 (5)	0.0409 (10)	
H9	0.6240	0.1848	0.8561	0.049*	
C10	0.5357 (4)	0.3159 (3)	0.7725 (5)	0.0384 (10)	

### Atomic displacement parameters (Å^2^)

**Table:**

	*U*^11^	*U*^22^	*U*^33^	*U*^12^	*U*^13^	*U*^23^
Cl1	0.0429 (6)	0.0487 (7)	0.0575 (7)	−0.0113 (5)	−0.0165 (5)	−0.0070 (6)
O1	0.0534 (19)	0.0280 (16)	0.067 (2)	0.0018 (14)	−0.0321 (16)	−0.0018 (15)
O2	0.059 (2)	0.0404 (18)	0.0501 (19)	−0.0077 (16)	−0.0147 (16)	−0.0133 (15)
O3	0.066 (2)	0.0288 (16)	0.067 (2)	−0.0032 (15)	−0.0280 (18)	−0.0044 (15)
O4	0.100 (3)	0.053 (2)	0.075 (3)	0.007 (2)	0.020 (2)	0.026 (2)
O5	0.051 (2)	0.071 (3)	0.126 (4)	0.018 (2)	−0.008 (2)	0.027 (3)
N1	0.0350 (18)	0.0245 (17)	0.0408 (19)	−0.0004 (14)	−0.0095 (15)	0.0022 (14)
N2	0.041 (2)	0.0329 (19)	0.045 (2)	−0.0018 (17)	−0.0123 (17)	0.0000 (17)
C1	0.0296 (19)	0.028 (2)	0.035 (2)	−0.0004 (16)	−0.0044 (16)	−0.0027 (16)
C2	0.035 (2)	0.027 (2)	0.035 (2)	−0.0060 (16)	−0.0063 (17)	−0.0014 (16)
C3	0.036 (2)	0.028 (2)	0.033 (2)	0.0013 (17)	−0.0020 (17)	−0.0014 (17)
C4	0.034 (2)	0.032 (2)	0.038 (2)	−0.0053 (16)	−0.0063 (17)	−0.0078 (17)
C5	0.050 (2)	0.027 (2)	0.050 (3)	−0.0077 (19)	−0.007 (2)	−0.0007 (19)
C6	0.044 (2)	0.026 (2)	0.048 (2)	0.0010 (18)	−0.012 (2)	0.0027 (18)
C7	0.032 (2)	0.028 (2)	0.043 (2)	0.0011 (17)	−0.0041 (18)	−0.0006 (17)
C8	0.040 (2)	0.028 (2)	0.041 (2)	0.0037 (18)	−0.0095 (18)	−0.0013 (18)
C9	0.040 (2)	0.039 (2)	0.039 (2)	0.0034 (19)	−0.0092 (19)	0.0011 (18)
C10	0.036 (2)	0.035 (2)	0.041 (2)	−0.0022 (18)	−0.0043 (19)	−0.0050 (18)

### Geometric parameters (Å, º)

**Table:**

Cl1—C4	1.729 (4)	C2—C3	1.383 (5)
O1—C7	1.239 (5)	C2—H2	0.9300
O2—C10	1.205 (5)	C3—C4	1.377 (5)
O3—C10	1.302 (5)	C4—C5	1.382 (6)
O3—H3O	0.82 (2)	C5—C6	1.386 (6)
O4—N2	1.209 (5)	C5—H5	0.9300
O5—N2	1.196 (5)	C6—H6	0.9300
N1—C7	1.340 (5)	C7—C8	1.479 (5)
N1—C1	1.413 (5)	C8—C9	1.326 (6)
N1—H1N	0.857 (19)	C8—H8	0.9300
N2—C3	1.469 (5)	C9—C10	1.497 (6)
C1—C2	1.389 (5)	C9—H9	0.9300
C1—C6	1.397 (6)		
			
C10—O3—H3O	110 (4)	C4—C5—C6	120.8 (4)
C7—N1—C1	126.7 (3)	C4—C5—H5	119.6
C7—N1—H1N	117 (3)	C6—C5—H5	119.6
C1—N1—H1N	115 (3)	C5—C6—C1	120.4 (4)
O5—N2—O4	123.9 (4)	C5—C6—H6	119.8
O5—N2—C3	118.7 (4)	C1—C6—H6	119.8
O4—N2—C3	117.5 (4)	O1—C7—N1	122.3 (3)
C2—C1—C6	119.2 (3)	O1—C7—C8	122.7 (4)
C2—C1—N1	123.8 (3)	N1—C7—C8	115.0 (3)
C6—C1—N1	116.9 (3)	C9—C8—C7	128.0 (4)
C3—C2—C1	118.6 (4)	C9—C8—H8	116.0
C3—C2—H2	120.7	C7—C8—H8	116.0
C1—C2—H2	120.7	C8—C9—C10	133.0 (4)
C4—C3—C2	123.1 (4)	C8—C9—H9	113.5
C4—C3—N2	120.2 (3)	C10—C9—H9	113.5
C2—C3—N2	116.7 (3)	O2—C10—O3	120.1 (4)
C3—C4—C5	117.8 (4)	O2—C10—C9	119.3 (4)
C3—C4—Cl1	122.4 (3)	O3—C10—C9	120.6 (3)
C5—C4—Cl1	119.8 (3)		
			
C7—N1—C1—C2	12.4 (7)	N2—C3—C4—Cl1	0.0 (6)
C7—N1—C1—C6	−167.4 (4)	C3—C4—C5—C6	1.4 (7)
C6—C1—C2—C3	3.9 (6)	Cl1—C4—C5—C6	−179.7 (4)
N1—C1—C2—C3	−176.0 (4)	C4—C5—C6—C1	0.3 (7)
C1—C2—C3—C4	−2.1 (7)	C2—C1—C6—C5	−3.0 (7)
C1—C2—C3—N2	178.4 (4)	N1—C1—C6—C5	176.8 (4)
O5—N2—C3—C4	57.7 (6)	C1—N1—C7—O1	−6.7 (7)
O4—N2—C3—C4	−123.7 (5)	C1—N1—C7—C8	173.9 (4)
O5—N2—C3—C2	−122.8 (5)	O1—C7—C8—C9	5.4 (8)
O4—N2—C3—C2	55.7 (5)	N1—C7—C8—C9	−175.2 (5)
C2—C3—C4—C5	−0.5 (7)	C7—C8—C9—C10	1.3 (9)
N2—C3—C4—C5	178.9 (4)	C8—C9—C10—O2	171.7 (5)
C2—C3—C4—Cl1	−179.4 (3)	C8—C9—C10—O3	−7.5 (8)

### Hydrogen-bond geometry (Å, º)

**Table:**

*D*—H···*A*	*D*—H	H···*A*	*D*···*A*	*D*—H···*A*
O3—H3*O*···O1	0.82 (2)	1.67 (2)	2.494 (4)	174 (7)
N1—H1*N*···O2^i^	0.86 (2)	2.01 (2)	2.839 (4)	162 (4)

Symmetry code: (i) −*x*+1, *y*−1/2, −*z*+3/2.
